# Venous revascularization to treat posterior nutcracker syndrome by transposition of the left gonadal vein: case report

**DOI:** 10.1590/1677-5449.190037

**Published:** 2019-09-30

**Authors:** Guilherme Lourenço de Macedo, Matheus Alves dos Santos, Andrey Biff Sarris, Ricardo Zanetti Gomes

**Affiliations:** 1 Universidade Estadual de Ponta Grossa – UEPG, Departamento de Medicina, Ponta Grossa, PR, Brasil

**Keywords:** nutcracker syndrome, hematuria, abdominal pain

## Abstract

The Nutcracker Syndrome is manifest in the presence of a symptomatic entrapment of the left renal vein between the abdominal aorta and the superior mesenteric artery. In a more ephemeral variation of this disorder, called the Posterior Nutcracker Syndrome, the renal vein is not compressed anterior to the aorta, but posteriorly, between the artery and the spine. Although there are multiple treatment options, current techniques aim to relieve the symptoms and reduce venous pressure on the left renal vein. This report describes a case of Posterior Nutcracker Syndrome in which the management approach chosen was open surgery, transposing the gonadal vein distally, to the inferior cava vein.

## INTRODUCTION

The nutcracker phenomenon is a rare anatomic entity, characterized by compression of the left renal vein (LRV) between the aorta and the superior mesenteric artery (SMA).^1^ When this formation causes clinical signs and symptoms, the nutcracker syndrome (NCS) is present. Less commonly, compression may occur between the aorta and the spinal column, when the LRV follows a retroaortic path, and such cases are called posterior nutcracker syndrome.^2^ The compression can cause venous hypertension with distal dilatation of the LRV and varicosities of the ureter and renal pelvis, manifesting with macroscopic and microscopic hematuria, flank pain and orthostatic proteinuria.^3^ Diagnosis is difficult, made on the basis of exclusion of other more prevalent causes, using imaging methods.^4^ Treatment varies, ranging from conservative management, through open surgery, to modern endovascular methods with stenting. The objective of the present study is to discuss the case of a young patient, with posterior NCS, who was treated with open venous revascularization surgery with transposition of the left gonadal vein (LGV).

## CASE DESCRIPTION

**Figure 1:**
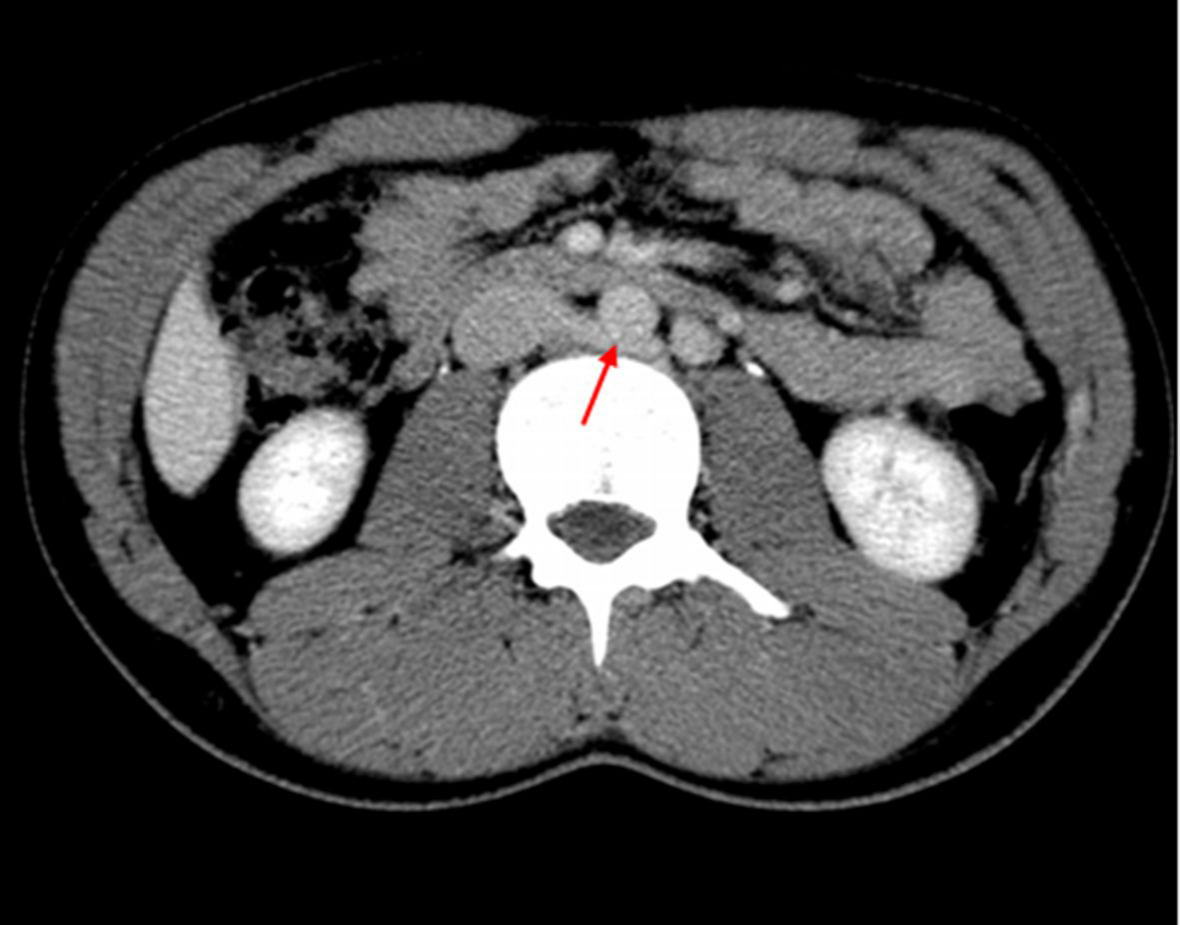
Abdominal computed tomography showing retroaortic compression of the left renal vein (arrow red). The patients’ symptomatic presentation combined with the imaging findings supported a diagnosis compatible with posterior nutcracker syndrome.

The patient was a 20-year-old male who presented at the medical service complaining of moderate to high intensity pain in the left flank. He described the pain as “stabbing” and stated that he had suffered from these pains since he was 6 years of age. They had initially been triggered by physical effort and had a frequency of one painful episode per week, but had become more recurrent, with sudden onset and without trigger factors (at rest) during the year preceding the consultation. He also reported an episode of macroscopic hematuria associated with pain one 1 week previously. He reported no other signs and no other complaints. He had no family history of nephropathy or hematuria. During physical examination he was lucid, well-oriented in space and time, with clear speech, good oxygenation, acyanotic, a heart rate of 84 bpm, blood pressure of 120 x 60 mmHg, and weight of 70 kg. Laboratory tests and initial clinical investigation were inconclusive. A computed tomography examination of the whole abdomen (Figure 1) revealed compression of the LRV between the aorta and spinal column and a dilated and tortuous LGV, with no pelvic varicose veins, confirming a diagnosis of posterior nutcracker syndrome. It was decided to manage the patient with clinical follow-up and he was medicated with diosmin-hesperidin and analgesics.

**Figure 2:**
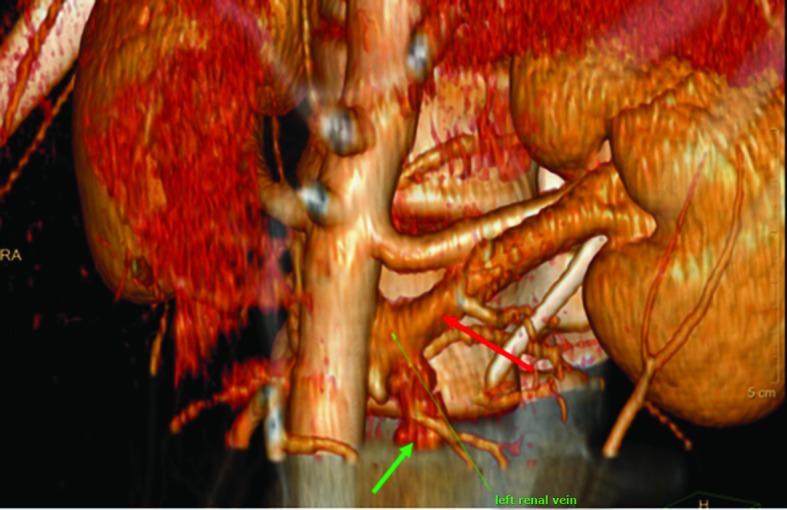
Angiotomography showing tortuosity (green arrow) of the left gonadal vein secondary to compression of the left renal vein (red arrow).

After a succession of return visits for further evaluations, the patient reported that the pain had not improved and there had been further episodes of macroscopic hematuria. Abdominal angiotomography (Figure 2) was ordered, to provide a better view of the compression and the decision was taken to conduct a surgical intervention for venous revascularization, without using a stent. The procedure chosen was transposition of the LGV, which was transected distally and reimplanted at the inferior vena cava (IVC), with a left paramedian incision and extraperitoneal access. The postoperative period was uneventful, the patient reported improvement of the painful complaints and the hematuria at later returns to the clinic, and he was discharged from the vascular surgery service.

## DISCUSSION

The first publication describing the nutcracker syndrome was written in 1950 by El-Sadr, who linked it to compression of the LRV along its path between the abdominal aorta and the superior mesenteric artery, caused by a narrowed angle between these two vessels, leading to varying degrees of obstruction of flow through the left renal vein, thereby provoking venous hypertension. The first description of surgery to treat the syndrome was published in the 1970s and since then variations of the technique for transposition of the renal vein have been described and have been reported as a an effective surgical method.^5^

The clinical manifestations of posterior NCS are similar to those of anterior NCS, with intermittent abdominal pains in the left flank and upper left quadrant, combined with hematuria and proteinuria triggered by exercise, while some patients may also develop pelvic hypertension with varicosities of the ureteral vein and left gonadal veins. Differential diagnosis includes nephrolithiasis, intrinsic glomerular disease, infection, trauma, hematuria related to exercise, polycystic kidney disease, tumors, renal vein thrombosis, benign prostate hyperplasia, and endometriosis.^6^

Diagnosis tends to be complicated and a hypothesis of the posterior form of the syndrome should be raised in patients with hematuria originating in the left kidney and with an anatomic variant of the left renal vein, after exclusion of other pathologies.^6^ In adults, computed tomography is the preferred method for investigating anatomic variants of the renal vein and the degree of compression, even if this requires subjecting them to a significant dose of radiation. The majority of patients discover the anomaly incidentally and it is important to be aware of it before a surgical procedure.^7^

Little more than ten cases of the posterior form of the syndrome have been described in the literature. The severity and stage of the symptoms and the age of the patient are essential to choice of the treatment used to reduce hypertension in the LRV.^8^ For patients under the age of 18, or those with mild hematuria, the best option is conservative treatment with at least 2 years’ follow-up.^4^ A number of different open surgery techniques have been used, of which transposition of the LRV is the most common procedure.^9^ Endovascular stenting can also be used, but it can be associated with adverse events such as stent migration, thrombosis, restenosis, deformities, and erosions.^8^ In the case of the patient described in this report, the choice to employ traditional surgical treatment was because the symptoms remained after clinical treatment and because of the risks involved with endovascular treatment, taking into account the age of the patient. Transposition of the gonadal vein was the technique chosen to treat this patient because it enables relief of the pelvic venous congestion and contributes to reducing the venous pressure in the LRV. The LGV is exposed via the transverse mesocolon. Its tributaries are ligated and the vein is isolated, transected distally and anastomosed to the IVC below the inferior mesenteric vein.^10^ The choice of technique was based on the shorter operating time, the lower volume of blood loss, and the fact that it avoids manipulation of the LRV during the operation.^11^ Despite this choice, there is limited experience with and results of this technique described in the literature, just as there are few reports of cases of posterior NCS.^1^

## CONCLUSIONS

Posterior NCS is characterized by compression of the LRV between the abdominal aorta and the spinal column. The syndrome is difficult to evaluate because of the very small number of reports on the entity in the literature and a high level of clinical suspicion is needed to achieve an early diagnosis and avoid unnecessary procedures and complications, such as thrombosis of the renal vein. Surgical treatment is indicated in cases of persistent hematuria with anemia, functional renal failure, and uncontrolled pelvic pain or if conservative treatment is ineffective after 2 years of clinical follow-up.
