# Supplementary material for: Venous revascularization to treat posterior nutcracker syndrome by transposition of the left gonadal vein: case report
Source: J Vasc Bras. 2019 Sep 30;18:e20190037. doi: 10.1590/1677-5449.190037 (PMC6788857; doi:10.1590/1677-5449.190037)
Supplement: Translation

# Translation: Revascularização venosa via transposição de veia gonadal esquerda em síndrome de *nutcracker* posterior: relato de caso

**Authors:** Guilherme Lourenço de Macedo, Matheus Alves dos Santos, Andrey Biff Sarris, Ricardo Zanetti Gomes

**Affiliations:** 1 Universidade Estadual de Ponta Grossa – UEPG, Departamento de Medicina, Ponta Grossa, PR, Brasil

**Keywords:** **Palavra-chave:** síndrome do quebra-nozes, hematúria, dor abdominal

## Abstract

A síndrome de *nutcracker* é manifesta na presença de um aprisionamento sintomático da veia renal esquerda entre a aorta abdominal e a artéria mesentérica superior. Uma variação mais efêmera desta desordem é dita síndrome de *nutcracker* posterior, quando a compressão da veia renal não mais ocorre frontalmente à aorta, mas posteriormente a ela, entre esta e a coluna vertebral. A despeito de variáveis opções terapêuticas, as técnicas presentes visam aliviar os sintomas e diminuir a pressão venosa da veia renal esquerda. Este relato descreve um caso de Síndrome de *nutcracker* posterior, em que a abordagem de escolha foi a cirurgia aberta, transpondo distalmente a veia gonadal esquerda na veia cava inferior.

## INTRODUÇÃO

O fenômeno de quebra-nozes é uma entidade anatômica rara, caracterizada pela compressão da veia renal esquerda (VRE) entre a aorta e a artéria mesentérica superior (AMS)^1^. Quando essa formação cursa com sinais e sintomas clínicos, tem-se a síndrome de quebra-nozes (SQN). Mais incomumente, a compressão pode ocorrer entre a aorta e a coluna vertebral, quando a VRE é retroaórtica, tratando-se, nesses casos, da síndrome de quebra-nozes posterior^2^. O quadro compressivo pode resultar em hipertensão venosa com dilatação distal da VRE, varizes ureterais e de pelve renal, manifestando-se como hematúria macro e microscópica, dor no flanco e proteinúria ortostática^3^. Seu diagnóstico é difícil, feito com base na exclusão de outras causas mais prevalentes, usando métodos de imagem^4^. O tratamento é variável, incluindo desde abordagens conservadoras e cirurgias abertas até modernos métodos endovasculares de colocação de *stent*. O presente estudo objetiva relatar e discutir o caso de um paciente jovem, com SQN posterior, cuja abordagem terapêutica escolhida foi a cirurgia aberta de revascularização venosa via transposição da veia gonadal esquerda (VGE).

## DESCRIÇÃO DO CASO

Paciente do sexo masculino, 20 anos, apresentou-se ao serviço médico com queixa de dor em flanco esquerdo de moderada a alta intensidade. A dor, descrita como sendo em “pontada” (sic), acompanhava o paciente desde os 6 anos. Inicialmente desencadeada por esforços físicos e na frequência de um episódio doloroso semanal, mostrou-se mais recorrente, de aparecimento súbito e sem fatores desencadeantes (ao repouso) no último ano que antecedeu a consulta. Associada à dor, queixava-se de episódio de hematúria macroscópica há 1 semana. Negava pródomos e outras queixas. Seu histórico era negativo para nefropatia e hematúria familiar. Ao exame físico, mostrava-se lúcido, orientado em tempo e espaço, eulálico, eupneico, afebril e acianótico, frequência cardíaca de 84 bpm, pressão arterial de 120 x 60 mmHg e peso de 70 kg. Os exames laboratoriais e a investigação clínica inicial foram inconclusivos. O paciente foi submetido à tomografia total de abdome (Figura 1), que demonstrou compressão da VRE entre artéria aorta e coluna vertebral, associada à dilatação e tortuosidade de VGE, sem presença de varizes pélvicas, firmando o diagnóstico de síndrome de quebra-nozes posterior. Optou-se por acompanhamento clínico, sendo o paciente medicado com diosmina-hesperidina e analgésicos.

**Figura 1:** Tomografia computadorizada de abdome denotando a compressão retroaórtica da veia renal esquerda (seta vermelha). O quadro sintomático no paciente, juntamente com a demonstração em método de imagem, permitiu o diagnóstico compatível com síndrome de quebra-nozes posterior.

Após sucessivos retornos para novas avaliações, o paciente referiu não haver melhora da dor e novos episódios de hematúria macroscópica. Solicitou-se angiotomografia de abdome (Figura 2) para melhor visualização de quadro compressivo e optou-se por intervenção cirúrgica de revascularização venosa sem uso de *stent*. O procedimento de escolha foi a transposição da VGE, que foi transeccionada distalmente e reimplantada na veia cava inferior (VCI), com incisão paramediana à esquerda e acesso extraperitoneal. O pós-operatório decorreu sem complicações, e o paciente relatou melhora das queixas álgicas e da hematúria nos posteriores retornos ambulatoriais, recebendo alta do serviço de cirurgia vascular.

**Figura 2:** Angiotomografia apontando (seta verde) a tortuosidade da veia gonadal esquerda secundária à compressão da veia renal esquerda (seta vermelha).

## DISCUSSÃO

A primeira publicação da síndrome de quebra-nozes foi feita em 1950 por El-Sadr, que associou a compressão da VRE no seu trajeto entre a aorta abdominal e a artéria mesentérica superior, causada pela diminuição do ângulo entre estes dois vasos, levando à obstrução, em graus variáveis, do fluxo da veia renal esquerda, provocando, assim, hipertensão venosa. A primeira descrição cirúrgica para o tratamento da síndrome foi proposta na década de 1970; desde então, variações da técnica de transposição da veia renal foram descritas e têm sido relatadas como método cirúrgico efetivo^5^.

As manifestações clínicas da SQN posterior são similares às da SQN anterior, como dor abdominal intermitente no flanco esquerdo e quadrante superior esquerdo associada à hematúria e proteinúria desencadeada por exercício, com alguns pacientes ainda podendo desenvolver hipertensão pélvica com varicosidades nas veias ureteral e gonadal esquerda. O diagnóstico diferencial inclui nefrolitíase, doença glomerular intrínseca, infecção, trauma, hematúria relacionada ao exercício, doença do rim policístico, tumor, trombose de veia renal, hiperplasia prostática benigna e endometriose^6^.

O diagnóstico costuma ser complicado, e a hipótese da forma posterior da síndrome deve ser levantada em pacientes com hematúria originada no rim esquerdo e com a presença de variação anatômica da veia renal esquerda, após excluídas outras patologias^6^. Em adultos, a tomografia computadorizada é o método preferido para investigar variações anatômicas da veia renal e o grau de compressão, mesmo que isso signifique expô-los a uma dose significativa de radiação. A maioria dos indivíduos descobre essa anomalia incidentalmente, sendo importante ter ciência disso antes do procedimento cirúrgico^7^.

Pouco mais de uma dezena de casos da forma posterior dessa síndrome foi descrita na literatura. A severidade e o estágio dos sintomas, além da idade do paciente, são essenciais para a escolha da modalidade de tratamento usado para reduzir a hipertensão da VRE^8^. Para pacientes menores de 18 anos ou aqueles que apresentam hematúria leve, a melhor opção de tratamento é a terapia conservadora com um acompanhamento de pelo menos 2 anos^4^. Diferentes técnicas de cirurgia aberta já foram realizadas, sendo a transposição da VRE o procedimento feito com mais frequência^9^. O *stent* endovascular também pode ser utilizado, porém, pode levar a adversidades como migração do *stent*, trombose, reestenose, deformidades e erosões^8^. No caso do paciente do presente relato, a opção pelo tratamento cirúrgico tradicional se deu por conta da persistência dos sintomas após abordagem clínica e pelos riscos do tratamento endovascular, levando em consideração a idade do paciente. A transposição da veia gonadal foi a técnica escolhida para o tratamento do paciente descrito, pois permite alívio da congestão venosa pélvica e contribui para a redução da pressão venosa na VRE. A exposição da VGE é feita via mesocólon transverso. Suas tributárias são ligadas e a veia é isolada, transeccionada distalmente e anastomosada na VCI abaixo da veia mesentéria inferior^10^. A opção pela técnica embasou-se no menor tempo operatório, na menor perda sanguínea e no fato de se evitar a manipulação da VRE durante o ato operatório^11^. Apesar da escolha, a experiência e os resultados com essa técnica são limitados na literatura, assim como relatos de caso da SQN posterior^1^.

## CONCLUSÃO

A SQN posterior é caracterizada pela compressão da VRE entre a aorta abdominal e a coluna vertebral. Devido ao escasso número de relatos na literatura sobre essa entidade, a síndrome apresenta dificuldades para sua avaliação e necessita de alto índice de suspeita clínica para obter um diagnóstico precoce e evitar procedimentos desnecessários e complicações, como a trombose da veia renal. O tratamento cirúrgico é indicado nos casos de hematúria persistente associada à anemia, insuficiência renal funcional e dor pélvica não controlada ou no caso de ineficácia de tratamento conservador após dois anos de acompanhamento clínico.
